# The role of hybrid FDG-PET/MRI on decision-making in presurgical evaluation of drug-resistant epilepsy

**DOI:** 10.1186/s12883-021-02352-z

**Published:** 2021-09-18

**Authors:** Márton Tóth, Péter Barsi, Zoltán Tóth, Katalin Borbély, János Lückl, Miklós Emri, Imre Repa, József Janszky, Tamás Dóczi, Zsolt Horváth, Péter Halász, Vera Juhos, Csilla Gyimesi, Beáta Bóné, Diána Kuperczkó, Réka Horváth, Ferenc Nagy, Anna Kelemen, Zsófia Jordán, Ákos Újvári, Koichi Hagiwara, Jean Isnard, Endre Pál, Attila Fekésházy, Dániel Fabó, Zsolt Vajda

**Affiliations:** 1 Department of Neurology, Medical School, University of Pécs, Rét u. 2, Pécs, H-7623 Hungary; 2 Department of Medical Imaging, Semmelweis University, Balassa út 6, Budapest, H-1083 Hungary; 3 Dr. József Baka Diagnostic, Radiation oncology, Research and Teaching Center, Somogy County Moritz Kaposi Teaching Hospital, Guba Sándor u. 40, Kaposvár, H-7400 Hungary; 4 MEDICOPUS Healthcare Provider and Public Nonprofit Ltd., Somogy County Moritz Kaposi Teaching Hospital, Guba Sándor u. 40, Kaposvár, H-7400 Hungary; 5 PET/CT Ambulance, National Institute of Oncology, Ráth György u.7-9, Budapest, H-1122 Hungary; 6 Division of Nuclear Medicine and Translational Imaging, Department of Medical Imaging, Faculty of Medicine, University of Debrecen, Nagyerdei krt. 98, Debrecen, H-4032 Hungary; 7 MTA-PTE Clinical Neuroscience MRI Research Group, Ifjúság u. 20, Pécs, H-7624 Hungary; 8 Department of Neurosurgery, Medical School, University of Pécs, Rét u. 2, Pécs, H-7623 Hungary; 9 National Institute of Clinical Neurosciences, Amerikai út 57, Budapest, H-1145 Hungary; 10 Epihope Non-Profit Kft, Szilágyi Erzsébet fasor 17-21, Budapest, 1026 Hungary; 11 Department of Neurology, Somogy County Moritz Kaposi Teaching Hospital, Sándor u. 40, Guba, H-7400 Hungary; 12 Epilepsy and Sleep Center, Fukuoka Sanno Hospital, 3-6-45, Momochihama, Sawara-ku, Fukuoka, 814-0001 Japan; 13 Department of Functional Neurology and Epileptology, Hospices Civils de Lyon, Hospital for Neurology and Neurosurgery Pierre Wertheimer, 59 Boulevard Pinel, 69500 Lyon, France

**Keywords:** Drug-resistant epilepsy, Hybrid FDG-PET/MRI, Clinical decision-making, Preoperative workflow, Epilepsy surgery

## Abstract

**Background:**

When MRI fails to detect a potentially epileptogenic lesion, the chance of a favorable outcome after epilepsy surgery becomes significantly lower (from 60 to 90% to 20–65%). Hybrid FDG-PET/MRI may provide additional information for identifying the epileptogenic zone. We aimed to investigate the possible effect of the introduction of hybrid FDG-PET/MRI into the algorithm of the decision-making in both lesional and non-lesional drug-resistant epileptic patients.

**Methods:**

In a prospective study of patients suffering from drug-resistant focal epilepsy, 30 nonlesional and 30 lesional cases with discordant presurgical results were evaluated using hybrid FDG-PET/MRI.

**Results:**

The hybrid imaging revealed morphological lesion in 18 patients and glucose hypometabolism in 29 patients within the nonlesional group. In the MRI positive group, 4 patients were found to be nonlesional, and in 9 patients at least one more epileptogenic lesion was discovered, while in another 17 cases the original lesion was confirmed by means of hybrid FDG-PET/MRI. As to the therapeutic decision-making, these results helped to indicate resective surgery instead of intracranial EEG (iEEG) monitoring in 2 cases, to avoid any further invasive diagnostic procedures in 7 patients, and to refer 21 patients for iEEG in the nonlesional group. Hybrid FDG-PET/MRI has also significantly changed the original therapeutic plans in the lesional group. Prior to the hybrid imaging, a resective surgery was considered in 3 patients, and iEEG was planned in 27 patients. However, 3 patients became eligible for resective surgery, 6 patients proved to be inoperable instead of iEEG, and 18 cases remained candidates for iEEG due to the hybrid FDG-PET/MRI. Two patients remained candidates for resective surgery and one patient became not eligible for any further invasive intervention.

**Conclusions:**

The results of hybrid FDG-PET/MRI significantly altered the original plans in 19 of 60 cases. The introduction of hybrid FDG-PET/MRI into the presurgical evaluation process had a potential modifying effect on clinical decision-making.

**Trial registration:**

Trial registry: Scientific Research Ethics Committee of the Medical Research Council of Hungary. Trial registration number: 008899/2016/OTIG. Date of registration: 08 February 2016.

## Background

Epilepsy is one of the most prevalent neurological diseases with an incidence of 0.4–1 ‰ and a prevalence of 0.4–1% [1, 2]. Approximately 23–30% of the patients are drug-resistant [3–6]. In these cases surgical resection constitutes the best therapeutic option towards achieving seizure freedom [7–11]. In 60–70% of the patients, noninvasive video-EEG monitor and cranial MRI can be conclusive regarding resective surgery without additional investigation(s). In the remaining proportion of the patients, invasive EEG (iEEG) exploration with intracranial electrodes (subdural or depth electrodes) plays a pivotal role in nonlesional drug-resistant epilepsy, or temporal or extratemporal lesional epilepsy with discordant electro-clinical results [12–16]. Epileptologists might have 4 reasonable options for patients with focal onset medically intractable epilepsy: (1) resective surgery without iEEG investigation, (2) iEEG exploration, (3) neuromodulation therapies such as vagus nerve stimulation (VNS) or deep brain stimulation (DBS), (4) giving new antiepileptic drug(s). Among these possibilities, resective surgery can achieve a decidedly higher seizure-freedom rate than the others [9–11]. When MRI fails to detect a potentially epileptogenic lesion, the chances of a favourable outcome after epilepsy surgery become significantly lower (from 60 to 90% to 20–65%) [10, 11]. Also in this workflow, FDG-PET/MRI coregistration can be utilized to guide a second look at MRI studies previously reported as nonlesional, thus underpinning decision-making [17]. Some epilepsy centers reported the role of PET/MRI coregistration was finding lesion(s) in nonlesional drug-resistant epilepsy patients [18–21]. Another option is the hybrid FDG-PET/MRI in preparation for epilepsy surgery in both lesional and nonlesional cases, providing additional sensitivity for detecting possible epileptic foci [22–24].

The aim of the present study was to investigate the possible effect of the results of hybrid FDG-PET/MRI on the decision-making by the epileptologist in both lesional and nonlesional drug-resistant epileptic patients. For this purpose, we selected two drug-resistant epileptic patient groups of the same size (either lesional or nonlesional cases), where the noninvasive video-EEG monitor and brain MRI were not conclusive regarding the resective surgery. We hypothesized that the results of hybrid FDG-PET/MRI may affect the initial judgement of the presurgical team: (1) the patient is eligible for iEEG exploration; or (2) eligible for resective surgery; or (3) the patient is not eligible for any further invasive procedure. Thus, in the present study, findings related to the alterations of decision-making were analyzed in detail with respect to imagery results (FDG-PET and MRI).

## Methods

### Subjects

We prospectively selected 60 adult patients (35 males, 25 females, mean age: 33.02, range:18–55 years) undergoing pre-surgical evaluation for drug-resistant, focal-onset epilepsy at two tertiary academic medical centers: (1) Department of Neurology, University of Pécs and (2) National Institute of Clinical Neurosciences, Budapest. Of the patients, all suffering from focal-onset epilepsy, 52 were right-handed and 8 were left-handed. Patients with previous history of epilepsy surgery were not included. Patients were divided into groups based on whether they were lesional (30 patients, for details please see Table 2) with discordant investigational results or nonlesional (30 patients, for details please see Table 1).

MRI examinations at this stage of presurgical evaluation were acquired on 3 Tesla scanners (3 T Magnetom TIM Trio, Siemens) and contained the following sequences: i) 2D T2-weighted axial turbospin-echo (TSE) (slice thickness 4.0 mm, TE/TR: 74/6450 ms, 120° flip angle, matrix 280 × 320 interpolated to 560 × 640, voxel size: 0.34 × 0.34 × 4 mm, scan duration 0:52 s); ii) 2D T2-weighted coronal TSE (slice thickness 3.0 mm, TE/TR: 93/6980 ms, 120° flip angle, matrix 280 × 320 interpolated to 560 × 640, voxel size: 0.34 × 0.34 × 4 mm, scan duration 1:45 min); iii) 2D T2-weighted coronal TSE fluid-attenuated inversion recovery (FLAIR) (slice thickness 3.0 mm, TE/TR: 123/9000 ms, 120° flip angle, matrix 192 × 256, voxel size: 0.86 × 0.86 × 3 mm, scan duration 3:36 min); iv) 3D sagittal isotropic Magnetization Prepared Rapid Acquisition Gradient Echo (MPRAGE) (slice thickness 0.98 mm, TE/TR: 2.53/1900 ms, 9° flip angle, matrix 256 × 256, voxel size: 0.98 × 0.98 × 0.98 mm, scan duration 8:50 min); v) 3D axial susceptibility weighted imaging (SWI) (slice thickness 1.5 mm, TE/TR: 20/27 ms, 15° flip angle, matrix 182 × 256, voxel size: 0.9 × 0.9 × 1.5 mm, scan duration 4:05 min); vi) 2D axial EPI diffusion-weighted imaging (DWI) (slice thickness 3.0 mm, TE/TR: 91/4800 ms, b-values 0, 500 and 1000 s/mm2, matrix 168 × 192, voxel size: 1.2 × 1.2 × 3 mm, scan duration 2:30 s) and vii) 3D axial time-of-flight (TOF) MRA (slice thickness 0.7 mm, TE/TR: 3.86/22 ms, matrix 202 × 384, voxel size: 0.58 × 0.58 × 0.7 mm, scan duration 6:12 s).

All patients signed a written consent approved by Scientific Research Ethics Committee of the Medical Research Council of Hungary (008899/2016/OTIG).

### Procedure and material

**Fig. 1:**
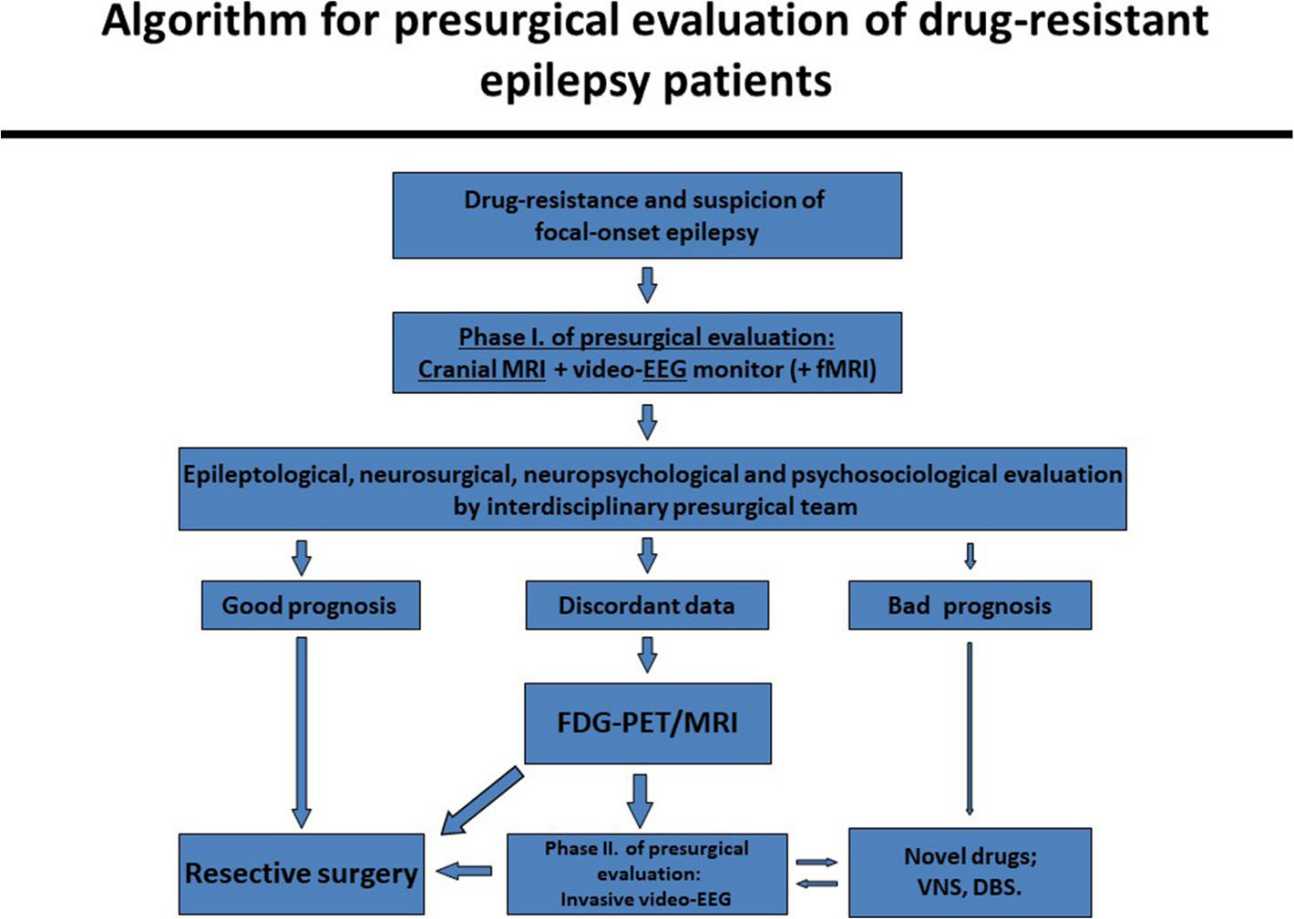
An algorithm describing the steps of presurgical evaluation at our centers

All of the patients underwent presurgical examinations: routine epilepsy clinic visits, noninvasive video-EEG monitoring, cranial MRI following the standard epilepsy protocol [25–28] and clinical semiology was evaluated by two presurgical teams. Before FDG-PET/MRI became available, some patients underwent PET/CT when MRI was negative or clinical and EEG findings suggested multiple seizure foci. An algorithm describing the steps of presurgical evaluation at our centers can be seen on Fig. 1. A total of 60 patients underwent pre-surgical evaluations with hybrid FDG-PET/MR from June 2016 until January 2018.

A hybrid FDG-PET/MRI system (Siemens Biograph mMR, Siemens Heathineers, Erlangen, Germany) consisting of 3 T Verio magnet and MR compatible LSO crystal based APD PET detector system allowing simultaneous PET/MRI acquisition was used. The device was settled in Baka József Diagnostic Center, Kaposi Mór Hospital, Kaposvár, Hungary.

The fluorine-18 fluoro-2-deoxyglucose ([18F] FDG) PET imaging was performed according to the guideline of European Association of Nuclear Medicine Neuroimaging Committee [29]. All patients fasted for at least 6 h before the scan and blood glucose levels were checked prior to FDG administration. Before the scanning procedure the patients were asked to empty their bladder and were positioned comfortably in a quiet, dark room equipped with a video camera. Then a cannula for intravenous administration was placed. A 2 h-long supervision of the patient and 30 min of video EEG recording was performed before the administration of FDG (bolus of 200 MBq i.v.). We maintained the video-EEG monitoring for the whole uptake phase of FDG to ensure interictal state. If any seizure activities were recorded during video-EEG monitoring, the FDG-PET/MRI investigation was postponed to the following day. The PET/MRI scan was started 60 min after the FDG administration. For the prevention of movement artifacts, we informed the patients that they should avoid voluntary movements in the scanner. After the scanning procedure the patients were further supervised for two half–lives of the radioisotope decay (ca. 240 min). To ensure complete simultaneous PET coverage, a 20 min and a 35 min list mode PET acquisition were applied. For PET attenuation correction purposes vendor-provided T2 ultrashort echo time (UTE) sequence was used, μMaps were generated automatically. From the PET RAW DATA a 20 min and a 35 min static image dataset were produced. Attenuation-corrected and uncorrected transaxial slices were generated. For PET image reconstruction OP-OSEM method was applied containing PSF correction (3 iterations, 21 subsets, 4 mm post-recon Gaussian filtering, 344 × 344 imaging matrix).

For MR imaging, the standard epilepsy protocol was used [25–28], comprising the following sequences: i) axial T2-weighted sequence used for PET attenuation correction (T2 UTE MRAC) (TR = 11.94 ms; TE1 = 0.07 ms, TE2 = 2.46 ms, flip angle = 10°; voxel size: 1.6 × 1.6 × 1.6 mm); ii) 2D T2-weighted axial TSE (slice thickness 4.0 mm, TE/TR: 106/6000 ms, 150° flip angle, matrix 358 × 448, voxel size: 0.5 × 0.5 × 4 mm, scan duration 4:08 s); iii) 2D T2-weighted coronal TSE (slice thickness 3.0 mm, TE/TR: 89/6770 ms, 150° flip angle, matrix 307 × 348, voxel size: 0.5 × 0.5 × 3 mm, scan duration 3:04 s); iv) 2D T2-weighted coronal TSE FLAIR (slice thickness 3.0 mm, TE/TR: 128/9000 ms, 120° flip angle, matrix 192 × 256, voxel size: 0.9 × 0.9 × 3 mm, scan duration 5:44 s); v) axial diffusion-tensor imaging (DTI) (slice thickness 4.0 mm, TE/TR: 95/3600 ms, matrix 128 × 128, voxel size: 1.7 × 1.7 × 4 mm, scan duration 3:59 s); vi) axial T2* (slice thickness 4.0 mm, TE/TR: 19.9/620 ms, 20° flip angle, matrix 205 × 256, voxel size: 0.4 × 0.4 × 4 mm, scan duration 2:09 s); vii) 3D T2-weighted sagittal gradient recalled echo (GRE) (slice thickness 1.0 mm, TE/TR: 409/3200 ms, 120° flip angle, matrix 261 × 256, voxel size: 0.5 × 0.5 × 1 mm, scan duration 4:43 s); viii) 3D T2-weighted sagittal GRE FLAIR (slice thickness 1.0 mm, TE/TR: 395/5000 ms, 120° flip angle, matrix 261 × 256, voxel size: 0.5 × 0.5 × 1 mm, scan duration 5:52 s) and ix) 3D sagittal MPRAGE (slice thickness 1.0 mm, TE/TR: 2.98/2300 ms, 9° flip angle, matrix 240 × 256, voxel size: 1.0 × 1.0 × 1.2 mm, scan duration 9:14 s).

MRI (both the prior studies and those with the accompanying PET) and PET studies were downloaded and blindly and separately re-interpreted, by two neuroradiologists (for MRI: PB and ZV) and nuclear medicine physicians (for PET: KB and ZT). The two MR studies were interpreted separately from each other by each of the neuroradiologists (PB, ZV)*.* Finally, simultaneously acquired MRI and PET images were evaluated on fused images, and clinical decisions were done with these data.

### New variables: MR status change, additional information provided by PET comparing to MRI and clinical decision according to PET/MRI results

#### MR status change

We created five new categories to describe the changes:

- nn: The patient was nonlesional prior to the study and he/she also remained nonlesional in this study.
- np: The patient was nonlesional prior to the study and changed to lesional in this study.
- nc: The patient was suspect for lesional prior to the study but the lesion was not confirmed in this study.
- pp.: The patient was lesional prior to the study and the study confirmed the original lesion.
- pp.+: The patient was lesional prior to the study and the study both confirmed the original lesion and found new epileptogenic lesion(s).

#### Additional information provided by PET comparing to MRI (indicating the localisation of glucose hypometabolism in nonlesional case as well as in lesional case, related to the MRI-lesion)

A. Positive, revealing unilateral area(s) of hypometabolism in a nonlesional case.

B. Positive, revealing bilateral area(s) of hypometabolism in a nonlesional case.

C. Positive, ipsilateral, related to the MRI-identified lesion in a lesional case.

D. Positive, ipsilateral, but not related to the MRI-identified lesion, pointing to new area(s) within the same hemisphere in a lesional case.

E. Positive, contralateral to MRI-lesion in a lesional case.

F. Positive, bilateral in lesional case in a lesional case.

G. Negative.

#### Clinical decisions according to PET/MRI results

The clinical decisions made by a consensus of the two multidisciplinary epilepsy surgery teams at two tertiary academic medical centers were classified into six categories:

1. : Remained as iEEG candidate.
2. : Resective surgery is available instead of iEEG.
3. : Considered as not eligible for any further invasive procedures instead of iEEG.
4. : Became iEEG candidate instead of resective surgery.
5. : Considered as not eligible for any further invasive procedures instead of resective surgery
6. : Remained as candidate for resective surgery.

### Statistical analysis

We applied the Chi-square independence test to determine if there is a significant relationship between “MRI status change” and “Additional information provided by PET comparing to MRI” and used Pearson residuals to highlight the most contributing between these category pairs. Because of the small sample size, we performed Fisher’s exact test to confirm the result of the Chi-square test.

## Results

### Nonlesional group

Hybrid PET/MRI examination revealed that 18 of 30 patients were found to have new specific epileptogenic MRI-lesion(s), while 29 of 30 patients had an abnormal FDG uptake. Two lesions were found in five patients. Hybrid PET/MRI helped to indicate resective surgery instead of iEEG monitoring in 2 patients due to congruent MRI, PET, semiological and EEG data; resective surgery was performed in both cases with Engel I outcome (for details, please see Table 1). In seven patients, hybrid PET/MRI enabled us to decide to avoid any further invasive diagnostic procedures due to multiple electroclinical and/or hypometabolic epileptic foci with MRI-negativity (6 patients) or negative imagery (both PET and MRI) results (1 patient). Of the remaining 21 patients referred to iEEG due to discordant electroclinical and PET/MRI results, in 11 cases one MRI-target was revealed; in 5 patients two potential epileptogenic lesions were found; while 5 cases remained nonlesional. In 3 of 11 patients of whom one MRI-target was revealed iEEG was performed concluding in resective surgery in 2 cases with Engel III and Engel IV outcome; the remaining case was considered as not operable and did not concede to perform DBS/VNS implantation.

**Table 1:** Detailed investigational results of nonlesional group

Patient No.	Age of epilepsy onset (years)	Age at investigation (years)	Time to presurgical evaluation (years)	Seizure type(s) and frequency	Localisation of epileptic focus according to clinical semiology and EEG findings	Neurocognitive deficit	Speech center lateralization defined by functional MRI	3 T MRI finding before the study	MRI findings in hybrid PET-MRI	FDG-PET/CT made before of our study (hypoMb)	FDG-PET findings in hybrid PET-MRI (hypoMb)	Additional information provided by PET comparing to MRI A, B, C, D, E, F, G	MR status change nn, np, nc, pp, pp+	Clinical decision according to PETMRI results	Outcome of iEEG and/or resective surgery outcome performed after our study	Time to resective surgery (years)
1.	16	26	10	Complex partial, 15/month, no GM	R temporal L temporal	Left hemisphere.	Left	nonlesional	nonlesional	Not performed	R temporal L temporoinsular L frontolateral	B	nn	3 - considered as not eligible for any further invasive procedures instead of iEEG	N/A	N/A
2.	2	22	20	Complex partial, 2–4/month, 1 GM/month	R frontal L frontal	Right hemisphere	Right	nonlesional	R cingular FCD	Not performed	R temporal L temporal L frontal	B	np	3 - considered as not eligible for any further invasive procedures instead of iEEG	DBS was implanted, Engel IV.	N/A
3.	18	44	26	Complex partial, 6/month, no GM	R frontal insular	Right hemisphere	Left	nonlesional	R temporopolar FCD	Not performed	R temporal R insular	A	np	1 - remained as iEEG candidate	iEEG was not yet performed.	N/A
4.	19	36	17	Complex partial, 2/month, no GM	L temporal insular	Bilateral.	Left	nonlesional	L HS, L precuneus FCD	Not performed	L temporal	B	np	1 - remained as iEEG candidate	iEEG and resective surgery was performed in 2016, Engel III outcome.	17
5.	18	27	9	Complex partial, 2/month, 1 GM/year	L frontal temporal	None.	N/A	nonlesional	Left collateral sulcus FCD	Not performed	L temporal	B	np	1 - remained as iEEG candidate	iEEG was not yet performed.	N/A
6.	32	34	2	Complex partial, 4/month, 1 GM/year	R temporal parietal	Right hemisphere	N/A	nonlesional	nonlesional	Not performed	R temporal	A	nn	1 - remained as iEEG candidate	iEEG was not yet performed.	N/A
7.	15	26	11	Hypermotor, 10/month, no GM	R frontal insular	None.	Right	nonlesional	nonlesional	Right frontal	negative	G	nn	3 - considered as not eligible for any further invasive procedures instead of iEEG	N/A	N/A
8.	6	28	22	Complex partial, 2/month, no GM	L frontal temporal	Bilateral.	N/A	nonlesional	L HS	Not performed	L temporal, L frontolateral	A	np	1 - remained as iEEG candidate	iEEG was not yet performed.	N/A
9.	10	43	33	Complex partial, 2/month, no GM	R temporal insular	Bilateral.	N/A	nonlesional	R HS, L HS	Not performed	R temporal, L temporoccipital	B	np	1 - remained as iEEG candidate	Patient did not concede to perform iEEG, DBS was implanted, Engel III.	N/A
10.	18	36	18	Complex partial, 1/month, no GM	L temporal	None.	Left	nonlesional	L temporopolar encephalokele	Not performed	L temporal	A	np	2 - resective surgery is avalaible instead of iEEG	Resective surgery was performed in Jan. 2018., Engel IA.	19
11.	17	43	26	Complex partial, 5/month, 1 GM/month	R frontal temporal insular	Right hemisphere	Left	nonlesional	R precentral gyrus FCD	Right frontotemporal	R frontocentral, R temporal	A	np	1 - remained as iEEG candidate	iEEG was not yet performed.	N/A
12.	12	22	10	Complex partial, 2/month, 1 GM/month	L frontal temporal	Left hemisphere.	N/A	nonlesional	L HS	Not performed	L temporal	A	np	1 - remained as iEEG candidate	iEEG was not yet performed.	N/A
13.	16	24	8	Complex partial, 30/month, 1 GM/year	L frontal temporal	Bilateral.	N/A	nonlesional	nonlesional	Not performed	R temporal R frontocentral L temporal L frontocentral	B	nn	1 - remained as iEEG candidate	iEEG was not yet performed.	N/A
14.	25	43	18	Complex partial, 1/month, no GM	R temporal insular L temporal	None.	N/A	nonlesional	L HS	Not performed	L temporal R temporal	B	np	1 - remained as iEEG candidate	iEEG was not yet performed.	N/A
15.	7	18	11	Complex partial, 15/month, 1 GM/year	L temporal parietal insular	Right hemisphere	Left	nonlesional	nonlesional	Not performed	R temporoparietal, R occipital, L temporoparietal, L occipital	B	nn	3 - considered as not eligible for any further invasive procedures instead of iEEG	N/A	N/A
16.	19	28	9	Complex partial, 25/month, no GM	L temporal insular	None.	N/A	nonlesional	nonlesional	Not performed	R temporal L temporal L frontomedial	B	nn	1 - remained as iEEG candidate	iEEG was not yet performed.	N/A
17.	3	26	23	Complex partial, 10/month, 1 GM/month	L frontal	Bilateral.	N/A	nonlesional	nonlesional	Not performed	R frontomedial R temporal L temporal	B	nn	3 - considered as not eligible for any further invasive procedures instead of iEEG	N/A	N/A
18.	10	46	36	Simplex partial sensomotor focal, 15/month, 1 GM/year	R frontal	Right hemisphere	N/A	nonlesional	R precentral gyrus FCD, L precentral gyrus FCD	Not performed	R parietal, L parietal	B	np	1 - remained as iEEG candidate	iEEG was not yet performed.	N/A
19.	15	39	24	Complex partial, 4/month, 1 GM/year	R frontal temporal insular L frontal temporal insular	Bilateral.	N/A	nonlesional	R posterior cingulate FCD	Not performed	R frontomedial	A	np	1 - remained as iEEG candidate	iEEG was not yet performed.	N/A
20.	10	28	18	Hypermotor, 12/month, 1 GM/month	L frontal insular	None.	N/A	nonlesional	nonlesional	Not performed	L frontal L temporoparietal	A	nn	1 - remained as iEEG candidate	iEEG was not yet performed.	N/A
21.	33	39	6	Complex partial, 1/month, no GM	R temporal L temporal	Bilateral.	N/A	nonlesional	R amygdalar FCD	Not performed	R temporal R insular	A	np	1 - remained as iEEG candidate	iEEG was not yet performed.	N/A
22.	32	39	7	Hypermotor, 15/month, no GM	L frontal temporal insular	Left hemisphere.	N/A	nonlesional	nonlesional	Not performed	L temporal L insular	A	nn	1 - remained as iEEG candidate	Patient did not concede to perform iEEG.	N/A
23.	3	38	35	Complex partial, 30/month, no GM	R frontal temporal parietal insular L frontal temporal parietal insular	Bilateral.	Left	nonlesional	L middle temporal sulcus FCD	Not performed	L temporal	A	np	1 - remained as iEEG candidate	iEEG was performed in 2016, resective surgery in 2017. 06., Engel IV outcome.	36
24.	4	18	14	Hypermotor, 30/month, no GM	R frontal temporal	Right hemisphere	Left	nonlesional	R anterior cingulate FCD, R straight gyrus FCD	Not performed	R frontolateral, R temporal	A	np	1 - remained as iEEG candidate	iEEG was not yet performed.	N/A
25.	17	37	20	Complex partial, 1/month, 1–3 GM/year	L temporal insular	Bilateral.	N/A	nonlesional	L HS	Not performed	L temporal	A	np	1 - remained as iEEG candidate	iEEG was not yet performed.	N/A
26.	7	18	11	Hypermotor, 24/month, no GM	L frontal	Left hemisphere.	Left	nonlesional	L superior frontal sulcus FCD	Negative	L frontolateral	A	np	2 - resective surgery is avalaible instead of iEEG	Resective surgery was performed in Jun. 2018., Engel I.	12
27.	20	42	22	Complex partial, 30/month, no GM	R frontal temporal L temporal	Left hemisphere.	Left	nonlesional	nonlesional	Left temporal	R temporal R insular L temporal	B	nn	1 - remained as iEEG candidate	iEEG was performed and and the patient was finally considered as not operable, Engel IV.	N/A
28.	8	18	10	Complex partial, 8/month, no GM	R frontal temporal L temporal	None.	N/A	nonlesional	nonlesional	Not performed	R frontal R temporoparietal L temporal	B	nn	1 - remained as iEEG candidate	iEEG was not yet performed.	N/A
29.	19	25	6	Hypermotor, 1/month, 1 GM/year	L frontal parietal	None.	N/A	nonlesional	L inferior insular FCD, L precentral sulcus FCD	Not performed	R temporal, R frontocentral	A	np	3 - considered as not eligible for any further invasive procedures instead of iEEG	N/A	N/A
30.	1	39	38	Hypermotor 2/month, 1 GM/month	R frontal insular L frontal	Bilateral.	N/A	nonlesional	nonlesional	Not performed	diffuse hypoMb	B	nn	3 - considered as not eligible for any further invasive procedures instead of iEEG	N/A	N/A

Assessing the data comparing the structural high-quality MRI from hybrid FDG PET/MRI (“MRI status change”) with the information providing the combination of glucose hypometabolism with structural data (“Additional information provided by PET comparing to MRI”), the Chi-square independence test and Fisher’s exact test showed a significant association (*p* < 10e-6 and p < 10e-10, respectively). The Pearson residuals suggested that the group “np” (the patients were nonlesional prior to the study and changed to lesional in this study) and group “A” (PET revealed unilateral area(s) of hypometabolism in nonlesional cases) have a strong positive association as well as between group “nn” (patients were nonlesional prior to the study and they also remained nonlesional in this study) and group “B” (PET revealed bilateral area(s) of hypometabolism in nonlesional cases).

### Lesional group

Two patients became eligible for resective surgery, because of concordant electroclinical and hybrid FDG-PET/MRI results, which confirmed the MRI-lesion visualized prior to this study. In one patient any further invasive investigation was found to be contraindicated due to discordant electroclinical and PET data; but more importantly, because of altered MRI-status (nonlesional).

iEEG was planned in 27 patients, of whom three patients became eligible for resective surgery because of concordant hybrid FDG-PET/MRI results and electroclinical data; resective surgery was performed with Engel I outcome in 1 case and Engel II outcome in 2 cases (for details, please see Table 2).

**Table 2:** Detailed investigational results of lesional group

Patient No.	Age of epilepsy onset (years)	Age at investigation (years)	Time to presurgical evaluation (years)	Seizure type(s) and frequency	Localisation of epileptic focus according to clinical semiology and EEG findings	Neurocognitive deficit	Speech center lateralization defined by functional MRI	3 T MRI finding before the study	MRI findings in hybrid PET-MRI	FDG-PET/CT made before of our study (hypoMb)	FDG-PET findings in hybrid PET-MRI (hypoMb)	Additional information provided by PET comparing to MRI A, B, C, D, E, F, G	MR status change nn, np, nc, pp, pp+	Clinical decision according to PETMRI results	Outcome of iEEG and/or resective surgery outcome performed after our study	Time to resective surgery (years)
31.	16	38	22	Hypermotor, 15/month, 1 GM/year	R frontal R temporal L frontal L temporal	Left hemisphere.	Left	L cingular FCD or PMG	L cingular PMG	Not performed	R temporal L frontomedial	F	pp	2 - resective surgery is avalaible instead of iEEG	Resective surgery was performed in Oct. 2018, Engel II.	23
32.	18	28	10	Complex partial, 30/month, 1 GM/year	L temporal L insular	None.	N/A	L HS	L HS	Not performed	L temporal	G	pp	2 - resective surgery is avalaible instead of iEEG	Resective surgery was not yet performed	N/A
33.	7	40	33	Complex partial, 8/month, 1 GM/year	R frontal R temporal R insular L frontal L temporal L insular	Bilateral.	Left	L and right insular FCD	L and right insular FCD	Not performed	R temporal R frontolateral R insular L temporal	F	pp	1 - remained as iEEG candidate	iEEG and resective surgery was performed in 2016, Engel II.	33
34.	10	38	28	Complex partial, 3/month, 1 GM/year	R frontal R temporal L frontal L temporal	Bilateral.	N/A	L cingular FCD susp.	nonlesional	Not performed	R hemispherial	A	nc	5 - considered as not eligible for any further invasive procedures instead of resective surgery	N/A	N/A
35.	3	29	26	Hypermotor, 45/month, no GM	R frontal R temporal	Bilateral.	Left	R frontolateral FCD	R sulcus frontalis superior FCD	Not performed	R frontomedial R temporal L temporal	F	pp	6 - remained as candidate for resective surgery	Resective surgery was performed in Jun. 2018., Engel I.	27
36.	21	48	27	Complex partial, 4/month, 1 GM/year	R frontal R temporal L temporal	None.	N/A	L temporal FCD, susp.	R frontopolar heterotopia	Not performed	R frontomedial R temporal L temporal	F	pp+	1 - remained as iEEG candidate	iEEG was not yet performed.	N/A
37.	13	27	14	Complex partial, 10/month, no GM	R frontal R temporal L temporal	None.	Left	L frontolateral heterotopia, susp.	nonlesional	Not performed	R temporooccipital L temporooccipital	F	nc	3 - considered as not eligible for any further invasive procedures instead of iEEG	N/A	N/A
38.	11	22	11	Complex partial, 8/month, 1 GM/month	L frontal L insular L parietal	Bilateral.	N/A	L insular ischaemia	L HS L insular ischaemia	Not performed	L temporoinsular	C	pp+	1 - remained as iEEG candidate	iEEG was not yet performed.	N/A
39.	17	38	21	Hypermotor, 4/month, 1 GM/year	R frontal R insular	None.	N/A	R frontolateral FCD	R frontolateral FCD, bilateral occipital heterotopia	Not performed	R frontal	C	pp+	1 - remained as iEEG candidate	iEEG was not yet performed.	N/A
40.	3	34	31	Complex partial, 20/month, 1 GM/month	L frontal L insular	Left hemisphere.	Left	L HS	L HS	Negatíve	L temporoinsular	C	pp	1 - remained as iEEG candidate	iEEG (2018.) and resective surgery was performed in 2020., Engel II.	34
41.	38	41	3	Complex partial, 30/month, no GM	L temporal	None.	N/A	L temporal pole FCD	L temporal pole FCD	Not performed	R temporal L temporal	F	pp	1 - remained as iEEG candidate	iEEG was not yet performed.	N/A
42.	4	33	29	Hypermotor, 1–2/month, no GM	L frontal L insular	Left hemisphere.	Left	L precentral-opercular FCD	L precentral-opercular FCD	Not performed	L frontolateral L insular	D	pp	1 - remained as iEEG candidate	iEEG (2017.) and resective surgery was performed in 2017., Engel I.	30
43.	13	48	35	Complex partial, 6/month, 1 GM/year	R frontal R temporal L temporal	None.	N/A	L collateral sulcus FCD	Bilateral collateral sulcus FCD	Not performed	R frontal R temporal R parietal L frontal L temporal L parietal	F	pp+	3 - considered as not eligible for any further invasive procedures instead of iEEG	N/A	N/A
44.	25	39	14	Complex partial, 4/month, no GM	L temporal	None.	Left	L parahippocampal gyrus FCD	L parahippocampal gyrus FCD	Not performed	L temporal	C	pp	6 - remained as candidate for resective surgery	Resective surgery was not yet performed.	N/A
45.	14	25	11	Complex partial, 1–2/month, 1–3 GM/year	L temporal	Left hemisphere.	Left	L temporopolar FCD	L temporopolar FCD	Not performed	L temporal	C	pp	1 - remained as iEEG candidate	iEEG (2017.) and resective surgery was performed in 2017., Engel I.	11
46.	19	21	2	Complex partial, 2/month, 1 GM/year	L temporal L frontal	Left hemisphere.	N/A	L fusiform gyrus FCD	L fusiform gyrus FCD	Not performed	L temporooccipital	D	pp	1 - remained as iEEG candidate	iEEG was not yet performed.	N/A
47.	32	49	17	Complex partial, 5/month, 1 GM/year	R temporal L temporal	Left hemisphere.	Left	L amygdalar FCD	L amygdalar FCD	Not performed	L temporal	C	pp	1 - remained as iEEG candidate	iEEG was not yet performed.	N/A
48.	19	26	7	Hypermotor, 2/month, 1 GM/month	R frontal	None.	Left	L hippocampal oedema	nonlesional	Not performed	R frontolateral R temporal R insular	A	nc	1 - remained as iEEG candidate	iEEG was not yet performed.	N/A
49.	2	23	21	Complex partial, 2/month, no GM	R frontal R temporal R parietal	None.	Left	R temporal pole FCD	R temporal pole FCD	Not performed	R temporal L temporal	F	pp	1 - remained as iEEG candidate	iEEG was not yet performed.	N/A
50.	3	30	27	Complex partial, 4/month, 1 GM/month	L temporal L frontal	Bilateral.	Left	L mesiobasotemporal FCD	L cingular FCD, L temporal FCD	Left temporal	L frontomedial L temporal	C	pp+	1 - remained as iEEG candidate	iEEG was performed in 2016, patient did not concede to perform neither resective surgery nor neuromodulation.	27
51.	19	38	19	Complex partial, 5/month, 1 GM/year	R frontal R temporal R parietal L frontal L temporal L parietal	Left hemisphere.	N/A	L HS	L HS L posterior cingulate FCD	Not performed	R temporal L temporoparietal	F	pp+	1 - remained as iEEG candidate	iEEG was not yet performed.	N/A
52.	14	44	30	Complex partial, 3/month, 1 GM/month	R frontal R temporal	Bilateral.	Left	R HS	R HS	Not performed	R temporal	C	pp	1 - remained as iEEG candidate	iEEG was not yet performed.	N/A
53.	14	24	10	Complex partial, 4/month, no GM	L frontal L temporal	None.	N/A	L hippocampal oedema	nonlesional	Not performed	R temporal L temporoparietal	B	nc	3 - considered as not eligible for any further invasive procedures instead of iEEG	N/A	N/A
54.	19	27	8	Complex partial, 8/month, 2 GM/month	L temporal	Left hemisphere.	Left	R collateral sulcus FCD	R collateral sulcus FCD	Not performed	L temporal	E	pp	1 - remained as iEEG candidate	iEEG (Febr. 2017.) and resective surgery was performed in Jun. 2017., Engel I.	9
55.	20	32	12	Complex partial, 8/month, no GM	R frontal R temporal L temporal	None.	N/A	R HS	R HS	Not performed	L hemispherial	E	pp+	3 - considered as not eligible for any further invasive procedures instead of iEEG	N/A	N/A
56.	18	29	11	Complex partial, 4/month, no GM	L temporooccipital L parietal	Bilateral.	Left	L HS	L parietotemporal PMG, heterotopia	Not performed	R temporoinsular L temporal	F	pp+	3 - considered as not eligible for any further invasive procedures instead of iEEG	N/A	N/A
57.	8	36	28	Complex partial, 7/month, 1 GM/year	R frontal L temporal	Bilateral.	Left	L HS	L HS	Not performed	R temporal	E	pp	3 - considered as not eligible for any further invasive procedures instead of iEEG	N/A	N/A
58.	6	20	14	Complex partial, 4/month, no GM	R frontal R temporoinsular	Bilateral.	Left	R temporal FCD	R HS, R uncus FCD	Not performed	R temporal R parietal	C	pp+	1 - remained as iEEG candidate	iEEG (2017) and resective surgery was performed in 2017, Engel II.	14
59.	21	57	36	Hypermotor, 8/month, no GM	R frontal R temporal	Right hemisphere.	Right	R frontolateral nodular heterotopia	R frontolateral nodular heterotopia	Not performed	R frontolateral hyperM	C	pp	2 - resective surgery is avalaible instead of iEEG	Resective surgery was performed in Nov. 2016, Engel II.	36
60.	18	32	14	Complex partial, 1/month, no GM	R parietal R insular L temporal L parietal L insular	None.	N/A	L HS	L HS	Not performed	L frontal L temporal	D	pp	1 - remained as iEEG candidate	iEEG was not yet performed.	N/A

Six of the remaining 27 patients proved to be inoperable instead of iEEG, 4 of these patients were found not to be confirmed lesional. In 3 patients, any further invasive procedure was contraindicated. Eighteen patients remained candidates for iEEG due to discordant electroclinical and FDG-PET/MRI results. In 6 of 18 patients iEEG was already performed concluding in resective surgery in all cases with Engel I (3 cases) and Engel II (3 cases) outcome.

Statistical analysis of the data comparing the structural high-quality MRI from hybrid FDG PET/MRI with the information providing the combination of glucose hypometabolism with structural data did not reveal strong association in any case in lesional group.

## Discussion

In the case of drug-resistant focal-onset epilepsy, the most important issue in the algorithm of the decision-making is to judge whether a drug-resistant epilepsy patient is eligible for (1) resective surgery, (2) iEEG monitoring, or, (3) not eligible for any further invasive procedures [7, 8].

PET/MRI coregistration has long been utilized in epilepsy centers and has been useful to guide a second look at MRI previously reported as nonlesional, thus guiding decision-making [17]. In an earlier publication, PET/MRI coregistration helped to find obvious lesion in 6 of 10 nonlesional drug-resistant epilepsy patients [18]. In a pediatric study, 31 consecutive pediatric nonlesional epilepsy patients were reported, of whom nine showed subtle pathologic abnormalities after second MRI-reading guided by PET/MRI coregistration [19]. In another study, 35 consecutive epilepsy patients with refractory focal epilepsy were investigated: structural MRI showed no lesion in 15 patients, of whom PET/MRI coregistration detected hypometabolism in 7 cases that was undetected on PET alone [20]. In a recent paper, 103 consecutive epileptic patients with FCD type 2 were reported, of whom 61 patients were lesional, while 42 cases were dubious or negative. The additional value of PET/MRI coregistration in these 42 patients was predominant, because MRI localized FCD type 2 in 35 of 42 patients [21].

Here we report the role of hybrid FDG-PET/MRI on the decision-making workflow. Thus, in the present study, findings related to the changes in possible decisions of the presurgical team were analyzed in detail with respect to separate imaging results (FDG-PET and MRI) in both lesional and nonlesional epilepsy patients.

Our main finding is that hybrid FDG-PET/MRI decidedly influenced the decision-making of the presurgical team significantly. Its cardinal effect was the increased sensitivity of brain MRI in 60% of nonlesional patients, which is a principal component in judging the chance for seizure-freedom (nonlesional cases: 20–65% vs. lesional cases: 60–90%) [10, 11].

In an earlier pilot study using hybrid PET/MRI, 11 epileptic patients were investigated by FDG-PET/MRI without gross structural abnormalities that could interfere with image processing. Unfortunately, it is not clear from this publication, whether or not new structural lesions were found [23].

In another pilot hybrid FDG-PET/MRI study, of the twenty-nine patients assessed who underwent epilepsy surgery evaluation, in four cases new structural MR lesions were detected with the aid of FDG-PET findings, and one patient showed a new abnormal hypometabolism without any MRI abnormality. All new FDG-PET/MR lesions were clinically significant with concordant EEG and/or SPECT results as potential epileptic foci [24]. Recently, the same research group reported that hybrid FDG-PET/MR identified new structural or functional lesions in 10 of 74 patients [22].

Our study aimed to clarify the possible role of hybrid FDG-PET/MRI on decision-making in drug-resistant partial-onset epilepsy patients as well as to compare this effect with earlier PET/MRI coregistration studies.

### Nonlesional group

In our study, the hybrid FDG-PET/MRI revealed a new morphological lesion in 18 patients and PET hypometabolism in 29 patients within the nonlesional group. In this patient cohort, PET revealed unilateral area(s) of hypometabolism. However, PET revealed bilateral area(s) of hypometabolism in those patients who were nonlesional prior to the study and also remained nonlesional in this study. These two correlations were proved to be statistically significant.

Due to hybrid FDG-PET/MRI results, resective surgery was indicated instead of iEEG monitoring in 2 cases with an Engel I outcome; hybrid FDG-PET/MRI results helped to avoid any further invasive diagnostic procedures in 7 patients. The remaining 21 patients were referred to iEEG. In 16 of the remaining 21 patients, novel specific epileptogenic MRI-lesion(s) were revealed, proposing potential target(s) for iEEG monitoring, hopefully increasing the chance of successful identification of the epileptogenic zone. Three of them were underwent iEEG and 2 of them resective surgery with Engel III and IV outcome.

As we showed in Methods section, (beyond better image quality/resolution) a major difference was the application of 3D FLAIR sequence which was lacking in the earlier imaging protocol. This might in part explain the newly identified specific epileptogenic lesions in a significant proportion of the nonlesional group. Nine of these 18 patients showed the newly detected lesions exclusively on the 3D FLAIR images. Another possible explanation is the growing body of experience of our neuroradiologists together with the PET-readings (and these data were new information for them) and thus their increased sensitivity. Finally, the quality of MRI images deriving from the new hybrid PET/MRI systems was much better. These factors combined might explain the 60% difference (18 patients/30 nonlesional patients).

**Fig. 2:**
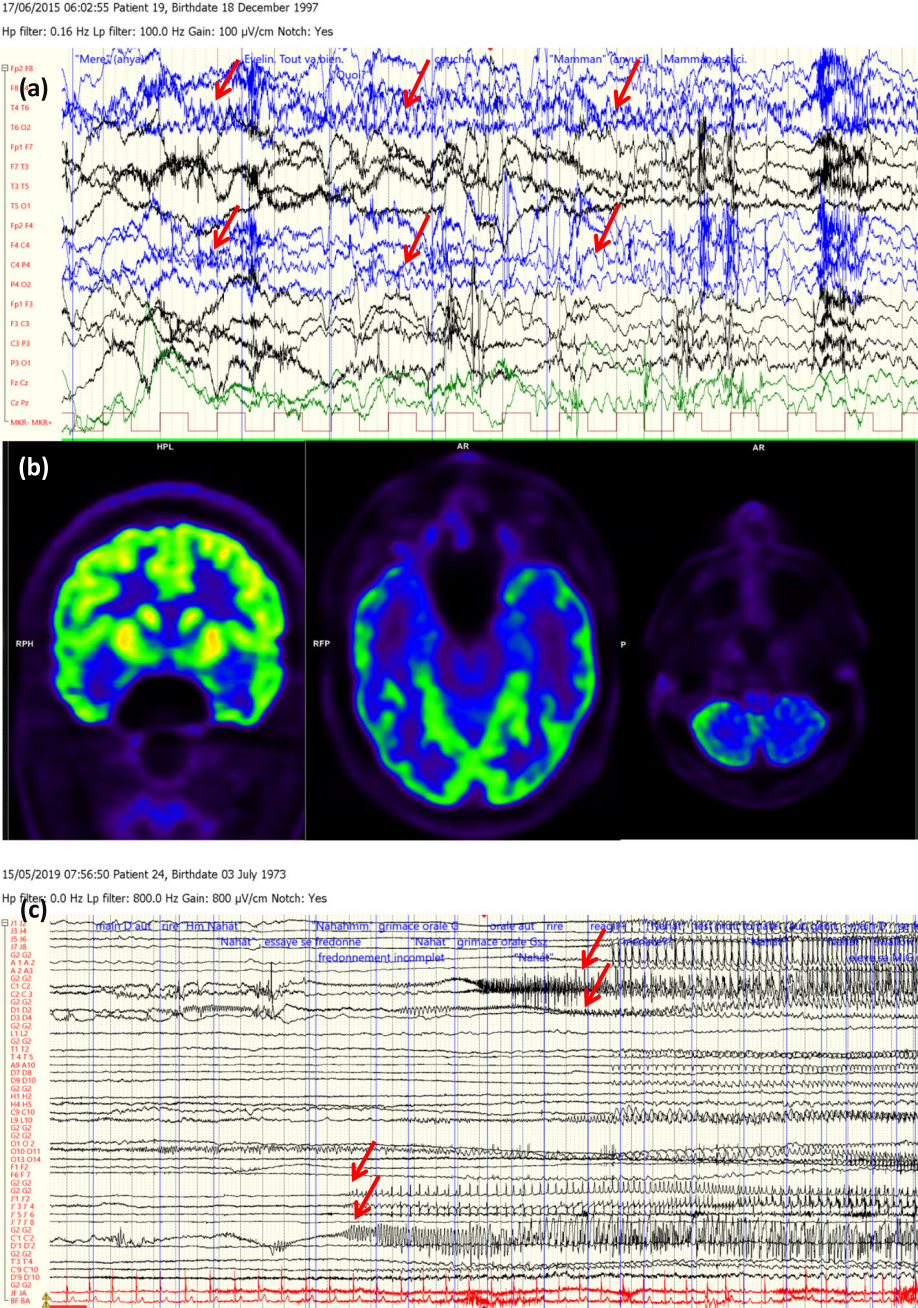
(Case 28, Table 1, group nn, decision type 1.): A drug-resistant epileptic patient with the electroclinical features of humming epilepsy. **a** Video-EEG monitoring: during one of his habitual seizure, a right frontotemporal seizure activity was registered (red arrows). Originally, he was nonlesional and this MRI-status did not change yet after this study. **b** and **c** 18F-FDG PET and PET/MRI presented a bitemporal hypometabolism with a right predominance (red boxes) and **d** a left cerebellar hypometabolism (red box). **c** This patient remained as an iEEG candidate. iEEG monitor has been performed and showed a bitemporal seizure activity with a left side onset (red arrows, left side of the figure), a left-right propagation in between a 10-s interval (red arrows, right side of the figure), which was remote-controlled by a possible left orbitofrontal seizure onset zone. The patient did not allow neither a second iEEG intervention, nor VNS or DBS implantation

**Fig. 3:**
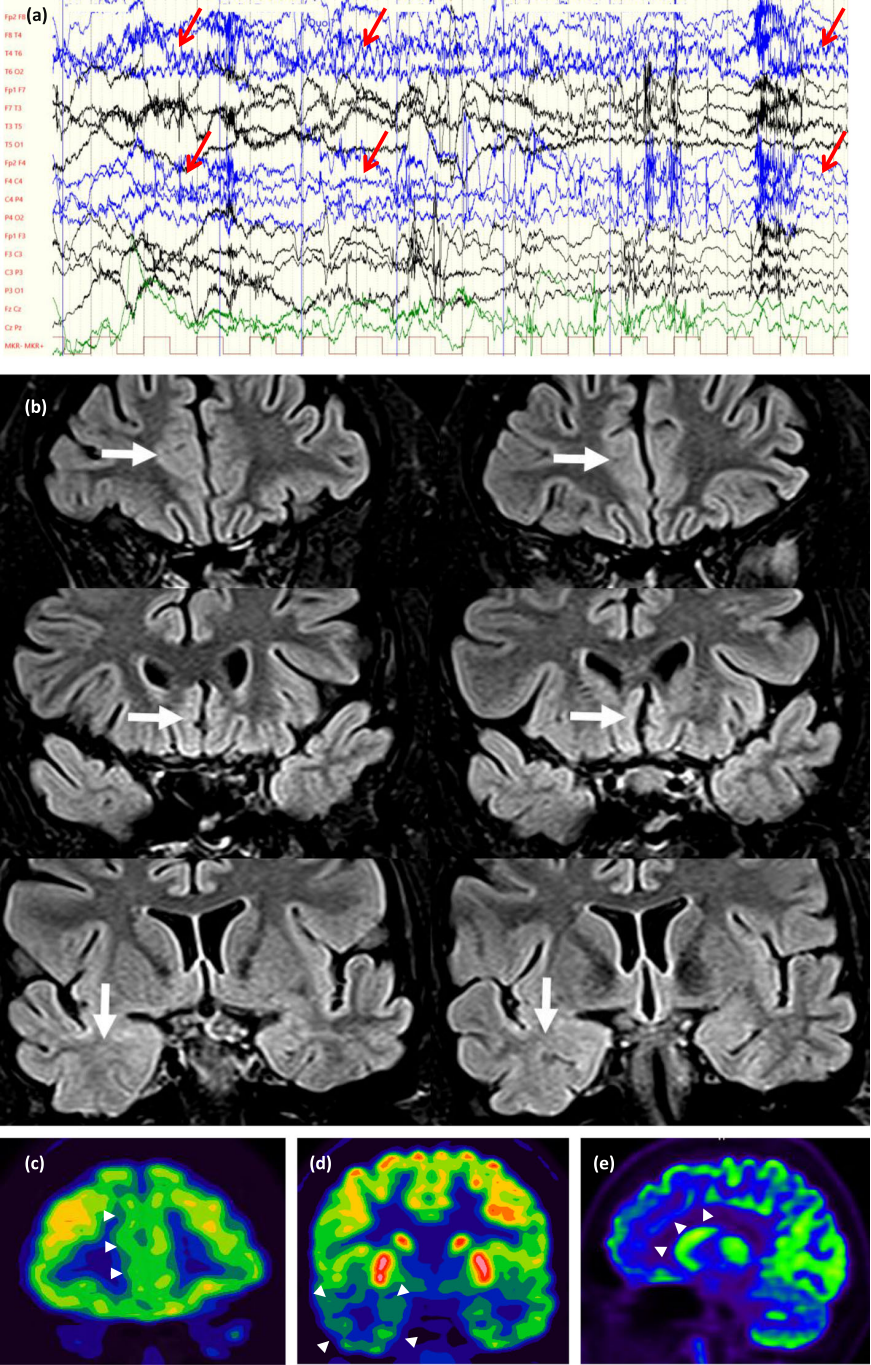
(Case 24, Table 1, group np, decision type 1.) A drug-resistant epileptic patient with the electroclinical features of a right frontotemporal epilepsy. **a** During video-EEG monitoring, her habitual hypermotor seizure with a right frontotemporal seizure activity was registered (red arrows). Originally, she was nonlesional and became lesional in this study. **b** Coronal FLAIR images: white arrows show possible focal cortical dysplasia in the right anterior cingulate cortex (upper row), in the medial cortex of the right straight gyrus (middle row), and the mildly increased signal intensity and blurred cortex-white matter interface in the right temporal lobe (lower row) can be seen. 18F-FDG PET and PET/MRI presented **c** and **e** a hypometabolism in the right mesiofrontal region (white arrowheads), **d** as well as in the right temporal lobe (white arroewheads). This patient remained as iEEG candidate; iEEG monitoring has not yet been realised

Moreover, in nine of the 30 cases, findings of hybrid FDG-PET/MRI resulted in a significant change in decision-making (iEEG monitoring, resective surgery or not eligible for any further invasive procedures) (Figs. 2 and 3).

### Lesional group

Hybrid FDG-PET/MRI disclosed at least one new morphological lesion in 9 patients and glucose hypometabolism in 30 patients within the lesional group.

Four patients found to be nonlesional in this study. In 17 cases the original lesion was confirmed. As we mentioned above, the major difference was the application of 3D FLAIR sequence which was lacking in the earlier imaging protocol, which might in part explain the newly identified specific epileptogenic lesions in the lesional group.

Hybrid FDG-PET/MRI has also significantly changed the original therapeutic plans in the lesional group. Three patients became eligible for resective surgery – two of them were operated with an Engel II outcome. Six patients were considered as not eligible for any further invasive procedure, and 18 cases still remained as candidates for iEEG. iEEG was already performed in 6 of 18 patients concluding in resective surgery with Engel I outcome in 3 cases and Engel II outcome in remaining 3 cases. Two patients remained as candidates for resective surgery - one of them was operated with an Engel I outcome. In both groups the new anatomical or functional lesions were found to be clinically significant.

**Fig. 4:**
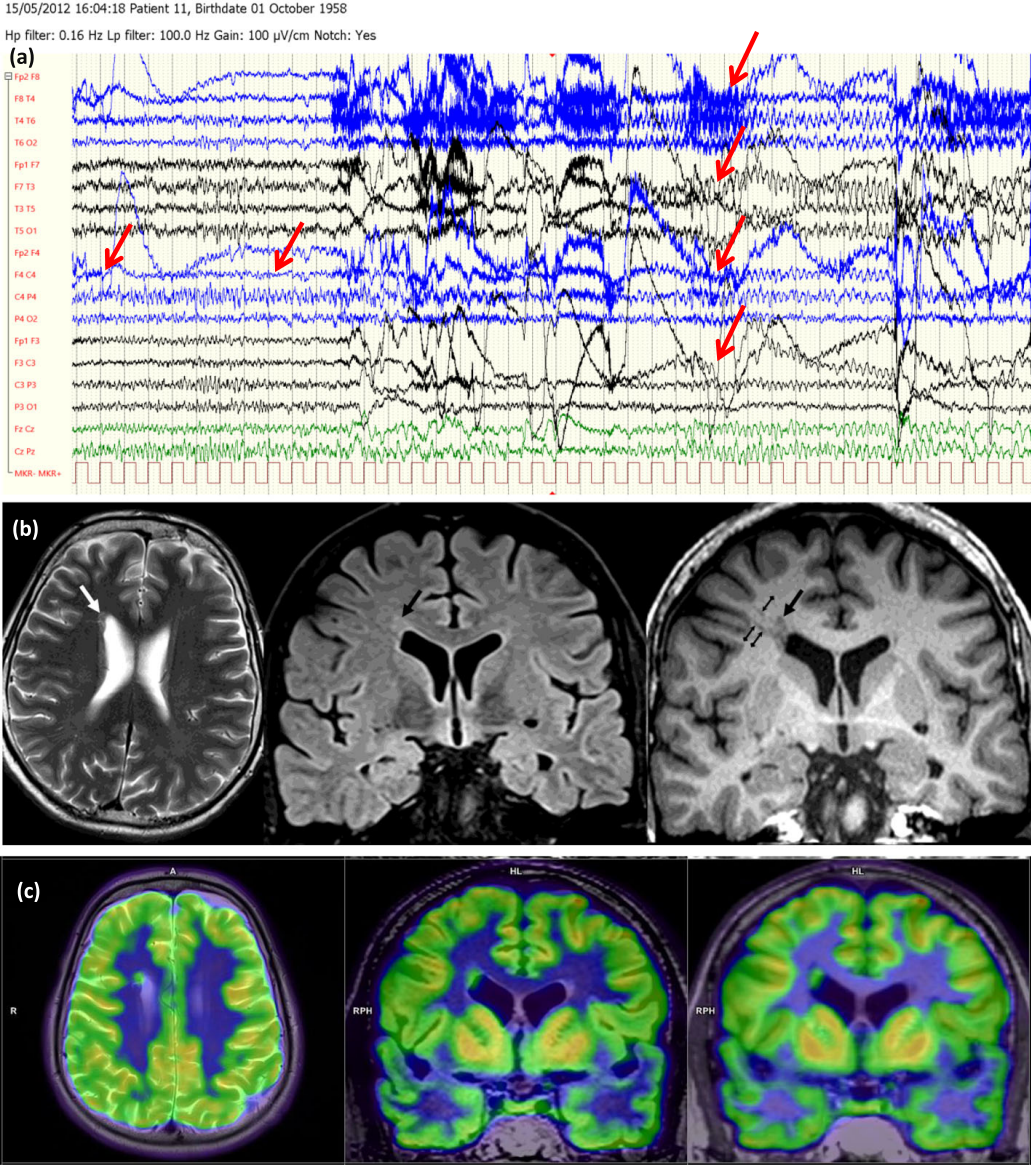
(Case 59, Table 2, group pp., decision type 2.): A drug-resistant epileptic patient with the electroclinical features of a right frontal epilepsy. **a** Video-EEG monitoring revealed his habitual seizure, a right frontocentral seizure activity was seen, which rapidly became bilateral (marked with red arrows). Concordantly, cranial MRI showed a nodular heterotopia in the right inferior frontal gyrus. **b** Axial T2 (left), coronal FLAIR (middle) and coronal T1 MPR (right) images. The white arrow on the T2 image and the large black arrows on the FLAIR and T1 images show focal nodular subependymal grey matter heterotopia. The small black arrows on the coronal T1 MPR image (right) show probable migrational bands. **c** Exceptionally compared to the other cases, during 18F-FDG PET and PET/MRI, a circumscribed FDG accumulation reaching the intensity of cortical tracer uptake (and highly exceeding white matter uptake) can be observed, identically to the right periventricular heterotopia. In this case, resective surgery became available instead of iEEG. Because the patient was left-handed, fMRI and also Wada-test were performed and they proved that in this case, active Broca region is localized in the right hemisphere. Thus, resective surgery was performed in awake state and finally, only a partial resection was possible. After the resective surgery, patient had much shorter (1–3 s long) seizures

**Fig. 5:**
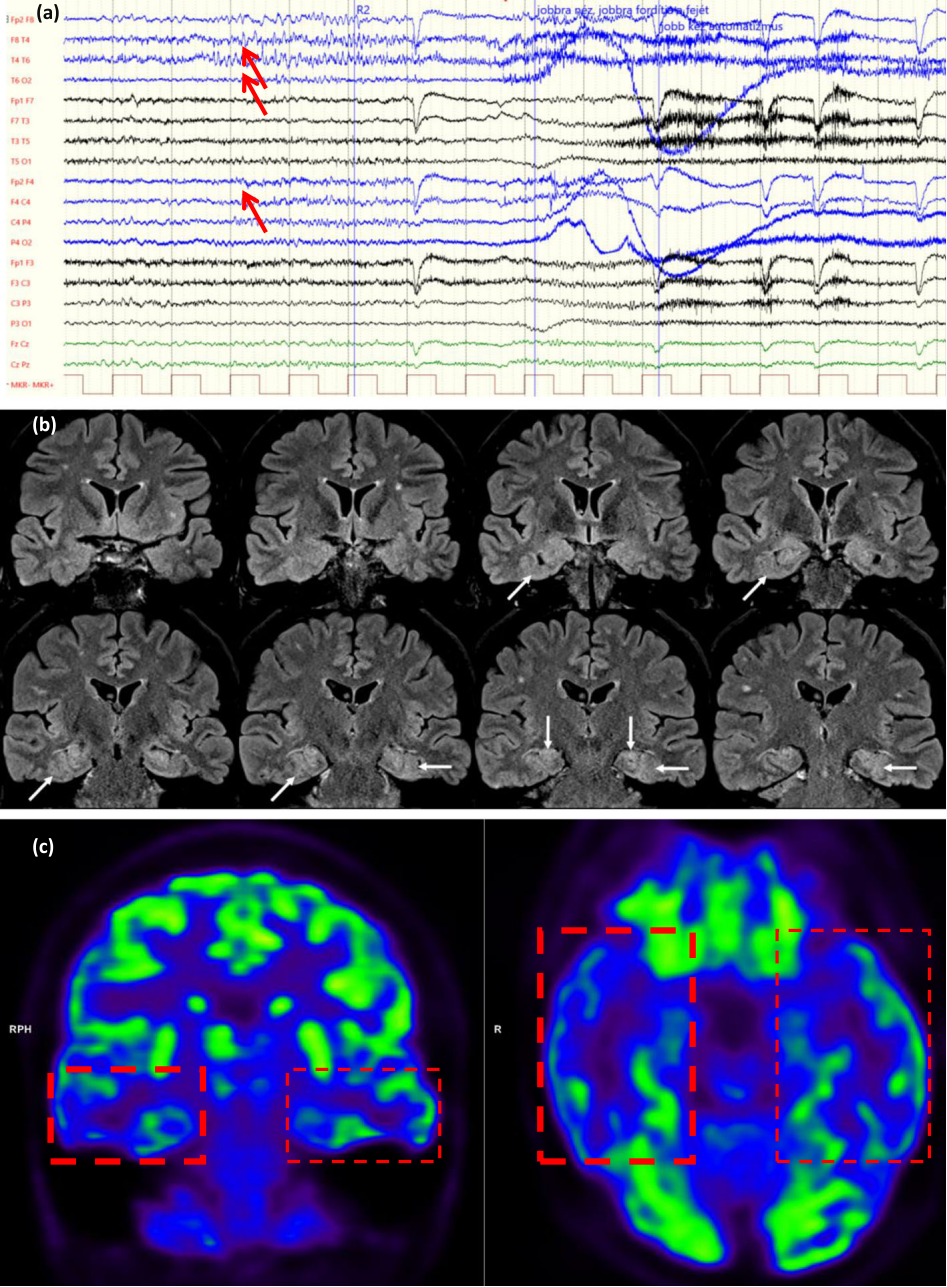
(Case 43, Table 2, group pp.+, decision type 3.) A drug-resistant epileptic patient with the electroclinical features of a bitemporal lobe epilepsy. **a** Video-EEG monitoring. During her habitual seizure, a right frontotemporal seizure rapid activity was seen (marked with red arrows). Meanwhile, original cranial MRI (made before this study) showed an FCD along the left collateral sulcus. **b** Cranial MRI made in this study (coronal FLAIR images): horizontal arrows show the originally detected FCD along the left collateral sulcus while the oblique arrows show the newly observed FCD along the right collateral sulcus. The vertical arrows show the typical configuration of bilateral hippocampal malrotation, while **c** and **d** 18F-FDG PET and PET/MRI presented a hypometabolism in the right and left frontotemporal lobe, with a right predominance (red boxes). In summary, this patient was considered as not eligible for any further invasive procedures instead of iEEG

In the lesional group, hybrid FDG-PET/MRI investigation altered the the epileptologist’s original decision in 10 of 30 cases: resective surgery, iEEG, or not eligible for any further invasive procedures (Figs. 4 and 5).

## Conclusions

Our study was undertaken to evaluate the potential improvement on decision-making using a hybrid FDG-PET/MRI scanner in epilepsy surgery algorithm, compared to separate 3 T MRI and electroclinical data. The results of hybrid FDG-PET/MRI significantly altered the original plans in 19 of 60 cases. In the nonlesional group, in 18 cases, novel specific epileptogenic MRI-lesions were revealed, proposing potential targets for iEEG monitoring, thus hopefully increasing the chance of successfully identifying the epileptogenic zone in the most difficult epilepsy patients cohort.

## Data Availability

The datasets used and/or analysed during the current study are available from the corresponding author on reasonable request.

## Abbreviations

DBS: Deep brain stimulation
DTI: Diffusion-tensor imaging
DWI: Diffusion-weighted imaging
[18F] FDG: Fluorine-18 fluoro-2-deoxyglucose
FLAIR: Fluid-attenuated inversion recovery
GRE: Gradient recalled echo
iEEG: Invasive EEG
MPRAGE: Magnetization Prepared Rapid Acquisition Gradient Echo
SWI: Susceptibility weighted imaging
TOF: Time-of-flight
TSE: Turbospin-echo
UTE: Ultrashort echo time
VNS: Vagus nerve stimulation
nn: The patient was nonlesional prior to the study and he/she also remained nonlesional in this study
np: The patient was nonlesional prior to the study and changed to lesional in this study
nc: The patient was suspect for lesional prior to the study but the lesion was not confirmed in this study
pp.: The patient was lesional prior to the study and the study confirmed the original lesion
pp. +: The patient was lesional prior to the study and the study both confirmed the original lesion and found new epileptogenic lesion(s)
A.: Positive, revealing unilateral area(s) of hypometabolism in a nonlesional case
B.: Positive, revealing bilateral area(s) of hypometabolism in a nonlesional case
C.: Positive, ipsilateral, related to the MRI-identified lesion in a lesional case
D.: Positive, ipsilateral, but not related to the MRI-identified lesion, pointing to new area(s) within the same hemisphere in a lesional case
E.: Positive, contralateral to MRI-lesion in a lesional case
F.: Positive, bilateral in lesional case in a lesional case
G.: Negative
1.: Remained as iEEG candidate
2.: Resective surgery is available instead of iEEG
3.: Considered as not eligible for any further invasive procedures instead of iEEG
4.: Became iEEG candidate instead of resective surgery
5.: Considered as not eligible for any further invasive procedures instead of resective surgery
6.: Remained as candidate for resective surgery
